# Clinical Cases

**Published:** 1868-01-15

**Authors:** 


### *CORRESPONDENCE.*

PHILADELPHIA, *January* 11*th*, 1868.

*Editor Chicago Medical Journal:*

In my last letter I gave you an account of an ovarian cyst, operated on by Prof. Wallace, in which he drew off the fluid and injected a solution of *Iodine*. The cyst did not seem to become filled with any fluid for four weeks, when it again commenced to enlarge, and it now has, apparently, about four pints in it, which quantity has been in it for about four weeks.

It has not, therefore, resulted in a *cure*, but it is certain that, thus far, very great relief has been afforded. I now proceed with my series of cases by giving you an account of

Mrs. A.; *æt.* 39. Presented herself at the clinic, with a tumor involving the parotid gland. An incision was made in the form of the letter S. Several small arteries were ligated; the tumor was divested of skin and superficial fascia, and the tumor wrung out with the hand. It involved the entire gland. The external carotid was ligated, and the portio-dura nerve was cut, which will of course produce paralysis of that side of the face. After a few hours the edges of wound were brought together and retained by several interrupted sutures, and a wedge-shaped compress applied. Patient was given a full opiate, and she rested well during the night. The wound was carefully dressed, and healed rapidly; she afterwards presenting herself at the clinic with the wound entirely healed, but with paralysis of right side of face. On examination by the microscope, the tumor was found to be made up of cancer cells.

Francis H.; *æt.* 9. *Case of Ectropia.* Had an abscess in the orbicularis muscle, which was punctured. In addition to this there were four ulcers about the eye, making it a complicated case. A V shaped piece of the conjunctiva was cut out, and the tarsal portion of the lid. The cellular tissue being adherent to the orbit, was loosened, and the attachments were all divided and broken up. The parts were now nicely brought together by delicate thread, and the hair-lip suture. Three weeks subsequently he presented himself with the lid very nearly perfect.

The following case presented itself to Prof. Wallace's "Obstetrical Clinic:"

Mrs. Fanny G.; *æt.* 42. Mother of two children. First noticed her womb "was down" four months ago, when it felt "very sore;" says she "often pushed it up." Examination shows prolapsus of vagina, with procidentia of uterus; a large sloughing ulcer covers the whole os and lips. The perineum has been ruptured, making the recto-vaginal bridge very limited, and the mouth of vagina very large, admitting with ease a large-sized fist. For three successive examinations, the ulcer was touched freely with *Arg. nit.*, and the uterus replaced, and Monsell's Solution freely applied over surface of vaginal walls, and then a colpeurynter introduced. This was dispensed with now, and the old cicatriced surfaces of the lacerated perineum were freshened with scissors, and then brought together nicely with quilled sutures. After this there was no infiltration of urine and no unpleasant symptom. She was afterwards shown to the class, her perineum firm and entire, and the uterus in good position, with ulcer healed.

Yours, truly, E. R. H.

